# Understanding the Role of Surface Modification of Randomized Trabecular Titanium Structures in Bone Tissue Regeneration: An Experimental Study

**DOI:** 10.3390/medicina58020315

**Published:** 2022-02-18

**Authors:** Elena Canciani, Vincenza Ragone, Carlo Alberto Biffi, Fabrizio Valenza, Riccardo D’Ambrosi, Matteo Olimpo, Aurora Cristofalo, Emanuela Galliera, Claudia Dellavia

**Affiliations:** 1 Department of Biomedical Surgical and Dental Sciences, Università degli Studi di Milano, 20133 Milan, Italy; aurora.cristofalo@studenti.unimi.it; 2 Research and Development Department, Permedica S.p.A., 23807 Merate, Italy; vincenza.ragone@gmail.com; 3 Consiglio Nazionale delle Ricerche, Istituto di Chimica della Materia Condensata e Tecnologie per l’Energia, CNR ICMATE, Unità Operativa di Lecco, 23900 Lecco, Italy; carloalberto.biffi@cnr.it; 4 Consiglio Nazionale delle Ricerche, Istituto di Chimica della Materia Condensata e Tecnologie per l’Energia, CNR ICMATE, Unità Operativa di Genova, 16149 Genova, Italy; fabrizio.valenza@cnr.it; 5 IRCCS Istituto Ortopedico Galeazzi, 20161 Milan, Italy; riccardo.dambrosi@hotmail.it (R.D.); emanuela.galliera@unimi.it (E.G.); 6 Department of Veterinary Sciences, Università degli Studi di Torino, 10095 Turin, Italy; matteo.olimpo@unito.it; 7 Department of Biomedical Sciences for Health, Università degli Studi di Milano, 20133 Milan, Italy

**Keywords:** osseointegration, bone, biological assay, histology, orthopedic, dental

## Abstract

*Background and Objectives:* Three-dimensional (3D) metallic trabecular structures made by additive manufacturing (AM) technologies promote new bone formation and osteointegration. Surface modifications by chemical treatments can improve the osteoconductive properties of metallic structures. An in vivo study in sheep was conducted to assess the bone response to randomized trabecular titanium structures that underwent a surface modification by chemical treatment compared to the bone response to the untreated specimens. *Material and Methods:* Sixteen specimens with a randomized trabecular titanium structure were implanted in the spongious bone of the distal femur and proximal tibia and the cortical bone of the tibial diaphysis of two sheep. Of them, eight implants had undergone a chemical treatment (treated) and were compared to eight implants with the same structure but native surfaces (native). The sheep were sacrificed at 6 weeks. Surface features of the lattice structures (native and treated) were analyzed using a 3D non-contact profilometer. Compression tests of 18 lattice cubes were performed to investigate the mechanical properties of the two structures. Excellent biocompatibility for the trabecular structures was demonstrated in vitro using a cell mouse fibroblast culture. Histomorphometric analysis was performed to evaluate bone implant contact and bone ingrowth. *Results:* A compression test of lattice cubic specimens revealed a comparable maximum compressive strength value between the two tested groups (5099 N for native surfaces; 5558 N for treated surfaces; *p* > 0.05). Compared to native surfaces, a homogenous formation of micropores was observed on the surface of most trabeculae that increased the surface roughness of the treated specimens (4.3 versus 3.2 µm). The cellular viability of cells seeded on three-dimensional structure surfaces increased over time compared to that on plastic surfaces. The histomorphometric data revealed a similar behavior and response in spongious and cortical bone formation. The percentage of the implant surface in direct contact with the regenerated bone matrix (BIC) was not significantly different between the two groups either in the spongious bone (BIC: 27% for treated specimens versus 30% for native samples) or in the cortical bone (BIC: 75% for treated specimens versus 77% for native samples). *Conclusions:* The results of this study reveal rapid osseointegration and excellent biocompatibility for the trabecular structure regardless of surface treatment using AM technologies. The application of implant surfaces can be optimized to achieve a strong press-fit and stability, overcoming the demand for additional chemical surface treatments.

## 1. Introduction

In recent years, the interest in additive manufacturing (AM) has been growing, thanks to the possibility of realizing complex and light 3D structures with potential bio-mimicry features, a high level of customization, and limited time to market [1,2,3]. AM can be an attractive technological solution, overcoming the limitations of conventional fabrication methods and being considered a potential alternative to the traditional manufacturing route, including casting, hot and cold plastic deformation, machining, and post-processing [4,5]. The orthopedic implants and devices sector can gain some advantages from the diffusion of AM techniques; the most relevant ones are realizing three-dimensional (3D) trabecular structures that mimic the bones’ trabecular structure and implant personalization for the patient [6]. Among AM methods, powder bed fusion processes, such as selective laser melting (SLM) and electron beam melting (EBM), offer great potential for the fabrication of high-quality porous metal implants [6]. In detail, SLM is the most diffused manufacturing technique used for producing complex and high-tech components, thanks to its superior precision in the realization of thin features [7,8,9,10]. In this regard, the SLM process has been deeply studied for the 3D printing of several alloys, such as Ti6Al4V, CoCrMo, and 316L, typically adopted in biomedical applications. Literature reports that a satisfactory porous structure should be open cellular and interconnected to ensure cell distribution and migration and facilitate blood vessel formation [11]. Due to the single-cell size, the minimum pore size must be approximately 100 μm, which allows the bone cells to migrate and be transported for osteogenesis [12,13]. The three-dimensional (3D) architecture and the characteristics of the surfaces of the prosthetic implants play a decisive role in the biological response. High porosity and an optimal pore size distribution are capable of promoting cell migration and vascularization. Randomly created irregular porous structures favor permeability and, therefore, the stimulation of bone growth within them.

Furthermore, the roughness of the surface structure stimulates the biological response in terms of proliferation and differentiation of osteoblasts, favoring an optimal bio-logical anchoring and the production of chemical factors necessary for the neovascularization and production of a new bone matrix [14]. It has been found that in addition to the 3D architecture, the surface of these implants can further improve bone osseointegration. Tsukanaka et al. conducted an in vitro study and observed that the etching treatment of the surfaces of trabecular titanium specimens significantly improves the differentiation of osteoblasts compared to the native surfaces [15,16]. These results were confirmed by in vivo studies showing that chemically treated trabecular titanium specimens have greater osteoconductive properties, which result in higher bone-to-implant contact (BIC) and more bone formation than untreated specimens [17,18]. The current study aimed to evaluate in vivo the response of the bone tissue to a randomized trabecular titanium structure with an increased micro-roughness surface compared to specimens with a native surface. In detail, the effect of the surface modification, performed using chemical etching on the osseointegration behavior of lattice structures in the Ti6Al4V alloy, manufactured through the SLM process, was investigated. Surface morphology, mechanical testing, and in vivo experimentation were carried out to establish the performances of the investigated chemical post processing.

## 2. Materials and Methods

### 2.1. Design of the Study

The study was designed with the aim to investigate the follow main object:

- Evaluation of a randomized trabecular titanium structure with the native surface versus a randomized trabecular titanium structure with the surface chemically treated, describing mechanical and morphological characteristics and cytocompatibility by means of an in vitro test and finally by a preclinical in vivo experiment in a sheep model.

To achieve these goals, a randomized trabecular titanium structure with a native surface (native surface) was compared to samples with the same 3D design and an increased micro-roughness surface obtained by chemical treatment (treated surface). To evaluate adhesion with cells, plane disks of smooth titanium (Ti smooth) and trabecular titanium with a native surface were produced (Ti trabecular).

The experimental plan was divided into the following steps:

- Sample preparation;
- Mechanical and morphological evaluation;
- In vitro biological assay;
- In vivo preclinical experiment.

### 2.2. Sample Preparation

#### 2.2.1. Randomized Trabecular Titanium Structure with the Native Surface

**Figure 1:**
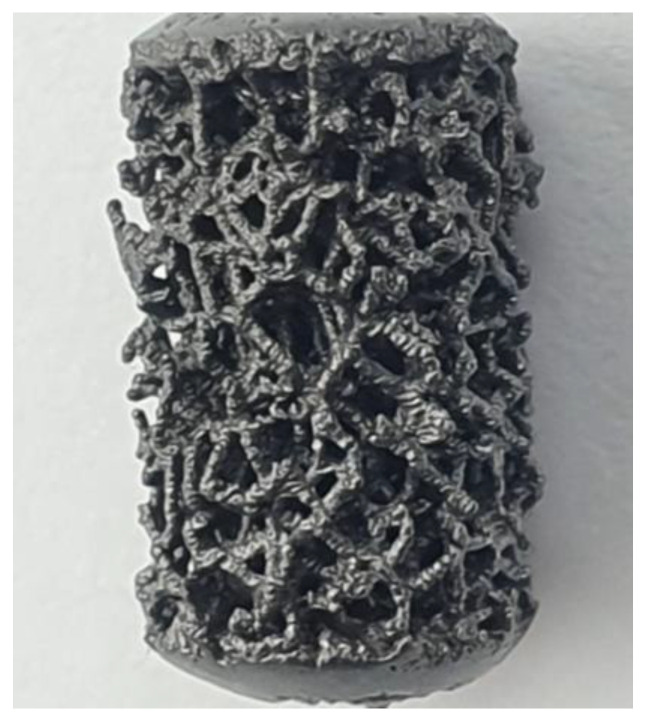
The macrophoto shows a 3D scaffold with a native surface. This prototype, 6.2 mm in diameter and 11 mm in length, was produced for the in vivo study experiment.

Based on the Voronoi tesselation method (a mathematic method to model bone-like three-dimension porous structures) (Figure 1), trabecular specimens were designed using the Autodesk® Within Medical software (Mill Valley, California, United States) [19,20]. Implants were produced by SLM technology (400 W Yb fiber laser, spot size of 70 µm, EOSINT M290 GmbH) using a commercially high-purity titanium metal powder with particle diameters of less than 54 µm. The samples were heat-treated at 800 °C for 4 h in an inert atmosphere and allowed to cool naturally to room temperature. The surface of trabecular implants was subsequently sand-blasted for 2–3 min using ceramics microsphere media to remove not-sintered particles from the lattice. Pore size distribution and porosity of the randomized trabecular structure ranged from 0.45 to 1.2 mm and 0.75% to 0.9%, respectively. The compressive modulus was between 1.1 and 1.7 GPa and comparable to the modulus of the human spongious bone.

#### 2.2.2. Randomized Trabecular Titanium Structure with an Increased Micro-Roughness Surface

In addition to the standard treatments to increase surface properties, a chemical treatment for surface modification was implemented. Specimens were treated in a hot mixture of acid HCl (36.5% Fluka), H_2_SO_4_ (95% JT Baker), and high-purity water (<1.1 μS/cm) at 80 °C for 10 min (H_2_O/HCl/H_2_SO_4_ 2:4:3 v:v). At the end of the chemical treatment, specimens were rinsed twice with ultrapure water in an ultrasonic bath.

### 2.3. Mechanical and Morphological Evaluation

#### 2.3.1. Scanning Electron Microscopy

Three samples per group (trabecular structure with native N3 and treated surfaces N3) were observed using a scanning electron microscope (SEM) (Jeol Neoscope Electron Microscope JCM-6000; Nikon, Tokyo, Japan). No additional fixation and previous coating of carbon film to evaluate the nano- and microstructure of the surface and the cleaning process were effectuated. Images were acquired at a total magnification of 22×, 70×, 650×, and 2000× and qualitatively assessed.

#### 2.3.2. Surface Roughness Characterization

Surface features of the lattice structures before and after chemical etching were quantitatively analyzed by the confocal technique using a 3D non-contact profilometer (Sensofar S-neox) equipped with two CF60-2 Nikon objectives, 20× and 100×, allowing measurements to be performed at different scales down to a vertical resolution of 8 and 2 nm and spatial sampling of 0.65 and 0.13 μm for the 20× and 100× objectives, respectively. In addition, quantitative measurements according to the ISO 25178:2016 standard were performed using the software embedded in the system (SensoSCAN). In particular, the surface roughness parameter, Sa, was chosen to account for the arithmetical mean height of the surface.

#### 2.3.3. Compression Test

The compression test of the two structures was investigated according to ISO 13314:2011. Eighteen cubes with a trabecular structure (side 15 mm) were tested for their mechanical proprieties. Nine of them had a native surface, and they were compared to nine cubes with an augmented roughness surface (treated surface). A compressive test was carried out at room temperature. Cubic specimens were displaced at a constant rate of 5 mm/min using an electromechanical testing apparatus with a 100 kN capacity (5982 Instron^®^, Norwood, MA, USA). A pressing device, consisting of a couple of polished parallel platens, was used to apply a compressive force to the test specimens. The maximum compressive strength reached during the compressive displacement was recorded in N.

### 2.4. In Vitro Biological Assay

#### 2.4.1. Cytotoxicity Test: In Vitro Evaluation

The cytotoxicity elution test (qualitative and quantitative evaluation) was conducted according to the EN ISO 10993-5 standard on a treated surface. A sub-confluence culture of a mouse fibroblastic cell line was used for the in vitro test. The extract was prepared as follows: the extract derived from the treated surfaces in submerged conditions was obtained with the complete cell culture medium incubated at 37 ± 1 °C for 24 h in a dynamic condition (ISO 10993-12). The extract was not filtrated. It was applied to a cell monolayer and incubated at 37 ± 1 °C in 5% CO_2_ for 24 h. A qualitative assessment was performed by observing the cells using an inverted microscope, and a quantitative evaluation was performed using the Neutral Red Uptake (NRU) method. NRU is a method that allows measuring cell viability by exploiting the ability of cells to incorporate and bind a cell viability dye, neutral red. This test was conducted to establish a safe manufacturing process of implants with an increased micro-roughness surface.

In addition, biocompatibility and cellular response were tested, culturing the cells on specimens with two different surfaces: a 2D smooth titanium surface (Ti smooth) and a 3D trabecular native surface of 1 cm × 1 cm × 0.3 cm (Ti trabecular). SAOS2 cells (ATCC^®^ HTB-85™ Homo sapiens bone osteosarcoma) [21] were seeded, and the effect of the surface and the 3D structure was evaluated by monitoring the changes in cellular viability compared to the viability of cells seeded on plastic (CNTR-). All experiments were performed in triplicate and on 2 consecutive days, at 37 °C in a 5% CO_2_ humidified incubator using DMEM containing 10% heat-inactivated fetal bovine serum (FBS) (GIBCO), supplemented by 100 U/mL penicillin (Invitrogen) and 100 μg/mL streptomycin (GIBCO) [3,4]. The vitality test was carried out at 3 time points (24, 48, and 72 h) using the AlamarBlue® test (Thermo Fisher Scientific Inc., Waltham, Massachusetts, United States), which allows evaluating metabolic function and cellular vitality by adding to the culture medium 10% indicator. The fluorescence was monitored at the 540 nm excitation wavelength, and a 580 nm emission wavelength was read using a GloMax Promega plate reader. For each group, a proliferation curve was created, and the percentage of the proliferation of cells at 24, 48, and 72 h was calculated referring to CNTR-, directly inserting the values in an algorithm, as described by the manufacturer’s protocol [22]. Finally, data were reported as the percentage mean and the standard deviation for each group.

#### 2.4.2. Cells Adhesion on the Implant Surface

SAOS2 (human primary osteogenic sarcoma) were cultured for 24 h on specimens, in triplicate, and treated for SEM analysis with Jeol Neoscope Electron Microscope (JCM-6000; Nikon, Tokyo, Japan) to assess the morphological adhesion of cells in contact with Ti smooth or Ti trabecular. In brief, the samples were fixed with 2.5% glutaraldehyde, post-fixed in osmium tetroxide, dehydrated through an increasing alcohol scale, infiltrated with hexamethyldisilazane, and finally dried overnight. Then the samples were mounted on stubs and coated with a thin layer of gold to allow image acquisition at the magnification of 1100×–1300× [22].

### 2.5. In Vivo Preclinical Experiment

#### 2.5.1. Surgical Procedure

The in vivo study was designed following the indications of ISO 10993-6:2016 and ISO 10993-2:2006 standards. This study used two skeletally mature sheep (Biellese breed) with ethical consent from the Italian Ministry of Health, General Direction of Animal Health and Veterinary Medicines (approval number 496/2017 PR, 16 June 2017). The sheep were bred for scientific purposes in accordance with the ethical code of practice relating to the care and use of animals in experiments. A total of 8 specimens (4 per animal) with an increased micro-roughness surface obtained by the implemented chemical protocol treatment (treated surface) were implanted in the right and left hind limbs and compared to the randomized trabecular titanium structure with the native surface. Overall, 16 implants were inserted into cylindrical defects drilled into the spongious bone of the distal femur and proximal tibia (*n* = 4 per animal) and the cortical bone of the tibial diaphysis (*n* = 4 per animal). Specimens had a cylindrical shape with a diameter of 6 mm and a length of 11 mm.

All implants were sterilized using an electron beam process irradiation before surgical implantation. A previously described implantation model with bilateral surgeries was used [20]. Before surgery, food and water were withheld for 24 h. In addition, animals were given subcutaneous carprofen (4 mg/kg) 12 h before surgery. Surgery was carried out in a dorsal decubitus position, so a gastric tube was placed to evacuate excess gas and fluids from the lattice-rumen during recumbence. Both hind limbs were shaved, disinfected, and draped free. To expose the bone, a 4 cm longitudinal incision was created on the lateral distal femur, the proximal medial tibia, and the medial mid-diaphysis tibia of both hind limbs. Soft tissue and periosteum were incised, and the periosteum was elevated over an area of about 2 cm^2^ to allow the implants’ insertion.

All wounds were closed in layers with 2.0 monofilament absorbable sutures and were covered with sterile dressings and soft bandages for 24 h. All animals were treated with the administration of oxytetracycline (9.5 mg/kg), cephalosporine (20 mg/kg), and diclofenac (2.3 mg/kg) for 7 days post operation. For all the implantations, a 6 mm diameter hole was created in the spongious bone using 3.2, 4.5, and 6 mm diameter drills in sequence. Irrigation with sterile saline was performed during drilling all bony defects. The 6 mm diameter implant was inserted in a press-fit manner using a custom-made impactor. Cortical holes were created using the same drill sequence used for the spongious bone. Holes in the tibial shaft were spaced approximately 2 cm apart to avoid stress concentrations and decrease the likelihood of fracture. Cortical implants were inserted in a line-to-line fashion, and holes were over-drilled to achieve the final dimension of implants. The sheep were free to move in their boxes and fully weight-bear until the animals were sacrificed and the implants were retrieved and processed for histologic and histomorphometric endpoints. The sheep were sacrificed, and specimens were harvested after 6 weeks. Implants (and the surrounding spongious or cortical bone) were isolated using a handheld saw.

#### 2.5.2. Histological Process

The samples were harvested and immediately fixed in formalin 10% without decalcification, dehydrated in an increasing concentration of ethanol (from 70% to 100%), and infiltrated in agitation in a vacuum in the dark using a solution of ethanol 100% and methacrylate until complete embedding in resin (Technovit 7200, Bio Optica, Milan, Italy). Each specimen was then polymerized using a polymerization machine Exakt 520 (Exakt Norderstedt, Germany) to create a solid block cut using a diamond blade (Micromet, Remet, Bologna). Next, each section was glued on the plastic slide (Exakt) with gluing resin (Technovit 7210, Bio Optica, Milan) and the gluing machine Exakt 402 (Exakt), ground to a thickness of 100 μm using a grinding system (LS2 Remet), and finally stained with toluidine blue/pyronin yellow for observation under a light optic microscope. Three representative transversal sections for each sample were observed to obtain a total of 48 sections that were digitally acquired at the total magnification of 400× using a high-resolution scanner (NanoZoomer S60, Hamamatsu) [20].

#### 2.5.3. Histological Assessment

- Qualitative evaluation of cellular and tissue reaction

The area surrounding each implant was analyzed on the histological sections acquired using a scanner at a high resolution (NanoZoomer S60, Hamamatsu, Japan). Morphological aspects indicated by ISO standard 10993 were evaluated through a digital pathology tool (NDP.view2 software, Hamamatsu, Japan). In particular, the presence of eventual local effects after implantation, such as neovascularization, fibrosis, necrosis, infiltration of fatty tissue, and inflammatory cellular response (polymorphonuclear cells, lymphocytes, plasma cells, macrophages, and giant cells), was evaluated [20].

- Histomorphometrical evaluation of osseointegration around the implant

The bone-to-implant contact (BIC) as a percentage of the surface of the implant in direct contact with the regenerated bone matrix and the bone in-growth (BIn) as the percentage of new bone matrix regenerated within the randomized trabecular titanium structure implant were calculated [20] to characterize the interface between the implants and the newly formed bone. The BIC is considered an essential marker for assessing osseointegration [23]. The BIC and the BIn were computed for each section on the whole scanned area using dedicated navigation software (NDP.view2 software, Hamamatsu, Japan) [20,23,24].

- Qualitative and semi-quantitative analysis of the regenerated area

The morphological features of the regenerated bone were qualitatively described separately for the cortical and spongious specimens to find features that characterized the regenerated area surrounding the randomized trabecular titanium structure (native or treated surfaces).

- Semi-quantitative analysis of the regenerated area

Morphological features of the regenerated bone were semi-quantitatively assessed separately for the cortical and spongious specimens for both groups (native and treated surfaces). A scoring system reported in a recent study [20] allows assigning a score ranging from 0 to 5 for quantifying the grade of regenerated tissue by evaluating the percentage of each tissue component (lamellar bone, woven bone, osteoid, and soft tissue) in the regenerated area around the randomized trabecular titanium structure in both groups. 

A score value between 0 and 5 (grade of regenerated tissue) was assigned to each tissue component as reported:

Grade 0: 0–0.9% 

Grade 1: 1–24% 

Grade 2: 25–39% 

Grade 3: 40–59% 

Grade 4: 60–79% 

Grade 5: 80–100%

### 2.6. Statistical Analysis

The experimental and control groups were compared using the inferential Student’s *t*-test for unpaired data, with a significance level of *p* < 0.05.

## 3. Results

### 3.1. Mechanical and Morphological Evaluation

#### 3.1.1. Implant Surface SEM

**Figure 2:**
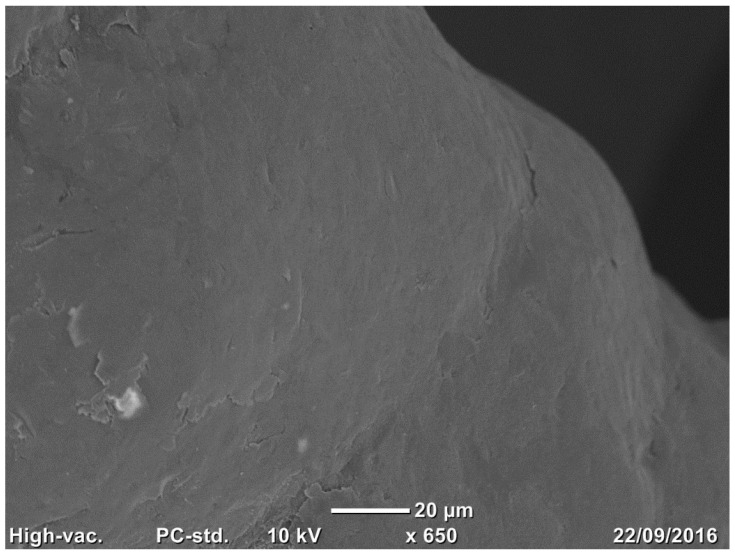
The microphoto shows the surface topography of the native surface that gained roughness through the sand-blasting process. SEM analysis, total magnification 600×.

**Figure 3:**
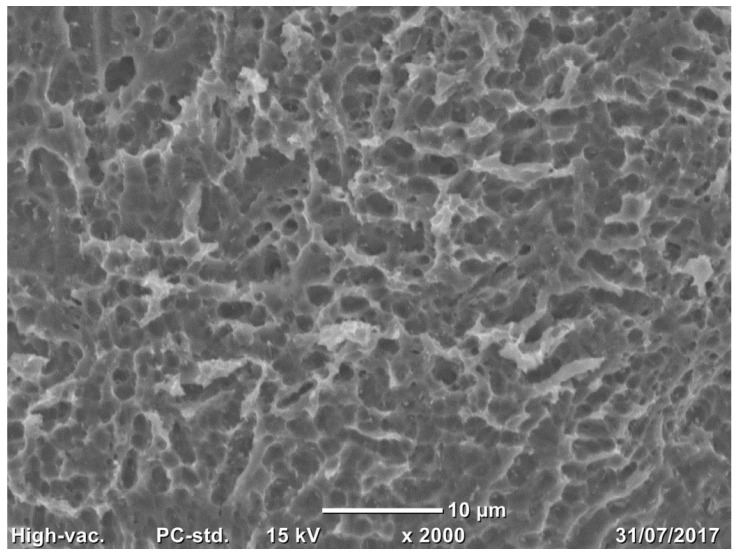
Small three-dimensional pores are observed on the surface of all trabeculae (range: 845 nm–11.2 µm) in the treated surface. SEM analysis, total magnification 2000×.

Compared to native surfaces (Figure 2), a homogenous formation of micropores was observed on the treated surface of most trabeculae due to chemical treatment (Figure 3).

#### 3.1.2. Profilometry

**Figure 4:**
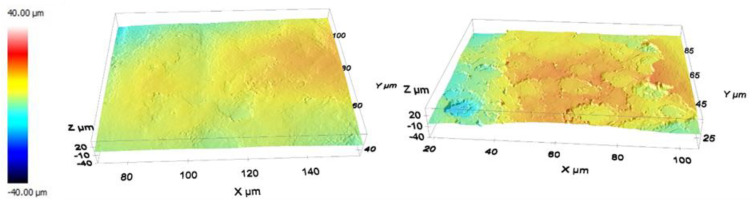
Three-dimensional reconstruction by a confocal microscope (objective 100×) of the native surface profile (**left**) and the treated surface (**right**). The color scale indicates the height of each point.

Observation of the 3D images under a confocal microscope (Figure 4) revealed that the treated surfaces had undergone etching by the chemical treatment. It resulted in an increase in the surface roughness, Sa, from 3.2 ± 0.6 µm to 4.3 ± 0.5 µm for the native and treated samples, respectively; measurements were performed over at least five small areas having a surface of 88 × 66 µm^2^.

#### 3.1.3. Compression Test

**Figure 5:**
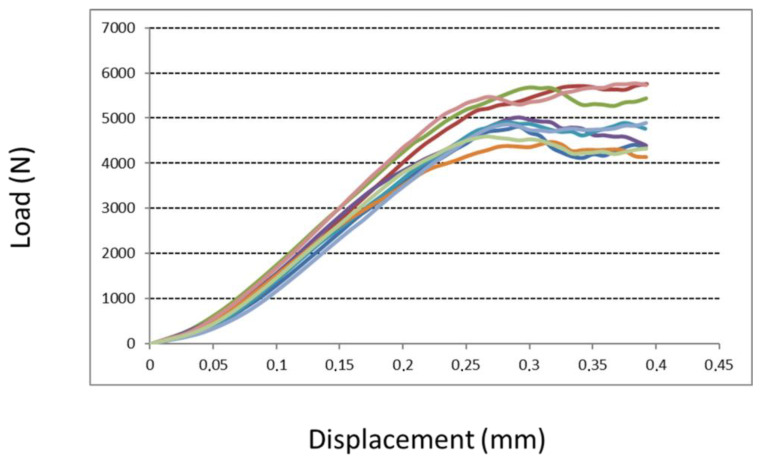
Compression test results for cubic specimens with native surfaces. The figure shows the increasing trend of the compression load (N) that the specimens faced before their failure: after a linear (elastic) trend, the load reaches its maximum value (peak) before the failure of the specimen. The displacement represents the deformation value of the specimen before it is crushed. All specimens failed within 0.4 mm of displacement. Each colored line represents the increasing trend of compression load of a specimen.

**Figure 6:**
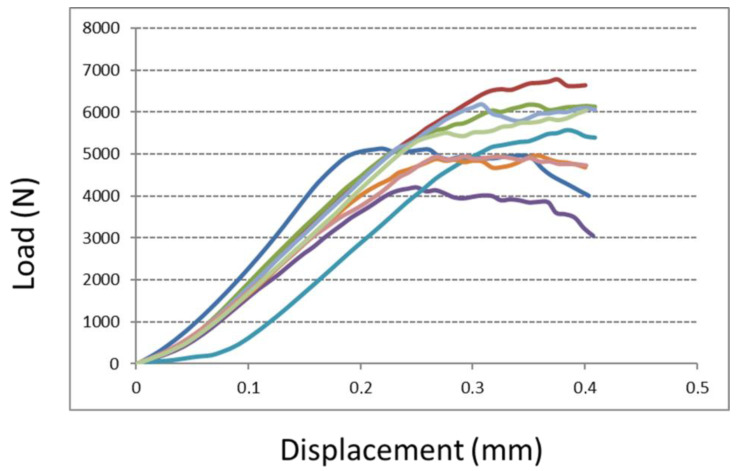
Compression test results for cubic specimens with treated surfaces. The explanation of the graphic is provided in Figure 5. Each colored line represents the increasing trend of compression load of a specimen.

A compression test of cubic specimens revealed a comparable maximum compressive strength value between the two tested groups (Figure 5 and Figure 6). Although a more considerable value was observed for specimen cubes with augmented roughness surfaces (treated surface), no significant differences were seen between the two groups (native versus treated surfaces). The maximum compressive strength for specimens with a native surface and with a treated surface was 5099 N (±502 N) and 5558 N (±811 N), respectively (*p* = 0.17).

### 3.2. In Vitro Biological Assay

#### 3.2.1. Cytotoxicity Test: In Vitro Evaluation

BalbC3T3 mouse fibroblast cells were seeded in 24-well culture plates, and after 24 h of incubation, the cells were observed under an inverted microscope to evaluate biological reactivity. After 24 h of contact, discrete intracytoplasmic granulations, no cell lysis, and no cell growth were observed in all treated cell cultures (degree of reactivity 0). In addition, cells treated with the extract did not show a reduction in cell viability (100%).

**Figure 7:**
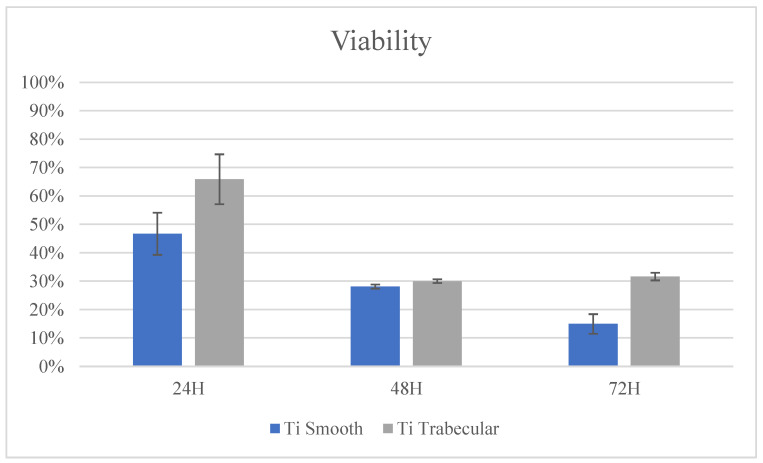
AlamarBlue^®^ test results. Axis-X shows time and axis-Y shows the percentage of incremented viability compared to CNTR data.

Cellular viability at 24, 48, and 72 h using the AlamarBlue^®^ test showed an increased vitality over time in all experimental groups. Both Ti smooth and Ti trabecular samples showed improved cellular viability after 24 h in culture compared to CNTR-. After 48 and 72 h in culture, the viability of cells seeded on both surfaces was higher than that of cells seeded on the CNTR- group, but it was lower than the values found at 24 h. Details are reported in Figure 7.

#### 3.2.2. Cells Adhesion on the Implant Surface

**Figure 8:**
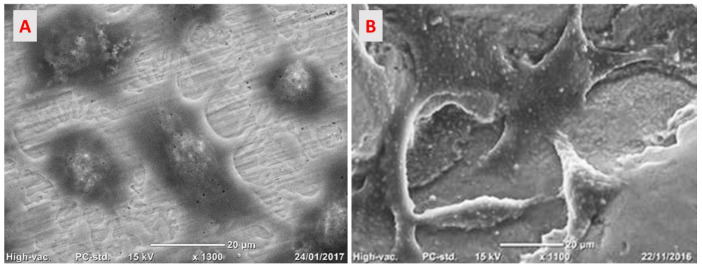
SAOS2 cells seeded on (**A**) the Ti smooth surface and (**B**) the Ti trabecular structure with a native surface. SEM analysis, total magnification of 1300×.

The cells seeded on the Ti smooth surface appeared spread, elongated, and enlarged. The cytoplasmic processes formed bridges of cell interconnection to stabilize the cells on the surface (Figure 8A). The cells on the Ti trabecular surface appeared to follow the surface, creating bridges through the cytoplasmic processes that seemed to join the titanium trabeculae following the three-dimensional structure (Figure 8B).

### 3.3. In Vivo Preclinical Experiment

#### 3.3.1. Histological Assessment

**Figure 9:**
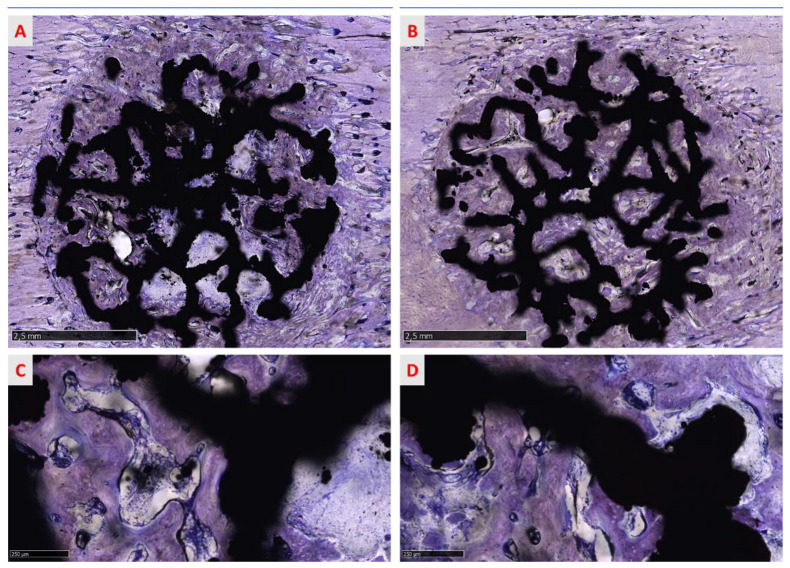
The panel shows overviews of two representative sections of (**A**) a trabecular structure with treated surfaces and (**B**) a trabecular structure with native surfaces per cortical bone. New compact bone surrounds the implant, englobing the specimens and filling the macropores of the implant. In both cases, the process of bone deposition does not seem wholly terminated and new lamellar bone seems in a phase of organization around the titanium trabeculae of the scaffold (**C**,**D**). Furthermore, newly regenerated bone appears well in contact and osseointegrated with the implant. Toluidine blue/pyronin yellow: (**A**,**B**) total magnification 13× and (**C**,**D**) total magnification 100×.

**Figure 10:**
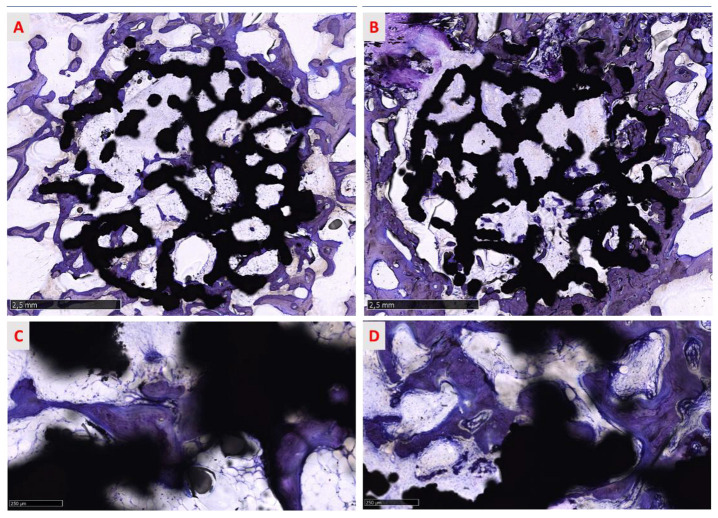
The panel shows the overviews of two representative sections of (**A**) a trabecular structure with treated surfaces and (**B**) a trabecular structure native surfaces per spongious bone. New bony trabeculae surround the titanium trabeculae, running into and among the macropores of the implant toward the implant surface (**C**,**D**). As reported for cortical bone, in both groups, the process of bone deposition is ongoing and new trabeculae are in a phase of maturation around the implant (**C**,**D**). Furthermore, newly formed bone comes into contact with the implant surface, osseointegrating with it. Toluidine blue/pyronin yellow: (**A**,**B**) total magnification 13× and (**C**,**D**) total magnification 100×.

All sites were histologically analyzed and the data obtained categorized by surface treatment (native and treated surface), type of bone (cortical and spongious bone), and evaluation of the response at 6 weeks (Figure 9 and Figure 10).

#### 3.3.2. Qualitative Evaluation of Cellular and Tissue Reaction

**Figure 11:**
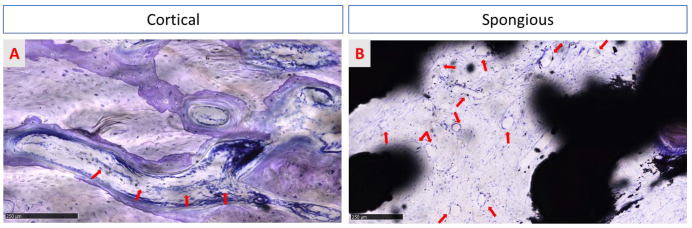
Both images show spaces occupied by the medulla. In treated surface samples, in cortical bone (**A**), neurovascular Haversian canals are characterized by blood vessels (red arrows), observable in longitudinal sections. In contrast, many vessels are detected in the medullary spaces of spongious bone (**B**) (red arrows). In both groups, no inflammatory infiltrate is observed. Toluidine blue/pyronin yellow: (**A**) total magnification 150× and (**B**) total magnification 100×.

Qualitative analysis of regenerated areas showed that there was no necrosis or inflammatory infiltrate in any site, but rare lymphocytes, plasma cells, and polymorphonucleates were observed (Figure 11A,B). Furthermore, no tissue reaction against the trabecular titanium structure was found either in the regenerated site or in the peripheral area of the implants in the native bone in both groups (treated and native samples). These data were confirmed by the absence of both fibrous tissue and ectopic fatty cells encapsulating the implants. The regenerated bone was found to be in close contact with the randomized trabecular titanium structure surface, without fibro-tissue interposition or gaps, as reported in Figure 9 and Figure 10. Furthermore, numerous blood vessels were detected in new Haversian canals of the cortical bone and in the medullary spaces of the spongious bone in the regenerated tissue that resulted in the active phase of the site regeneration. New vessels were organized in groups of 4–7 capillaries supported by a fibrous network of connective tissue (Figure 11B) in medullary spaces.

#### 3.3.3. Histomorphometric Evaluation of Osseointegration around the Implants

At 6 weeks after surgery, osseointegration was analyzed through BIC and BIn evaluation and their correlation. The mean and standard deviations in the BIC and BIn of the two groups (treated and native surfaces) were calculated separately in both cortical and spongious bone. The results show a different regenerative behavior, faster in compact bone than in spongious bone. Further, Table 1 shows no statistical differences between the two groups. BIC values in cortical and spongious bone are observed even if the data appear to be higher in the native surface group in both cases (*t*-test for unpaired data; *p* < 0.05). Indeed, these data are confirmed by BIn values, indicating that no significant differences in biology were observable for cortical bone and spongious bone (*t*-test for unpaired data; *p* < 0.05). However, the regeneration capacity around the native surface titanium implant and into the macropores of the randomized trabecular titanium structure seems higher than those in the treated surface group. Detailed results are reported in Table 1.

#### 3.3.4. Qualitative Analysis of the Regenerated Area

**Figure 12:**
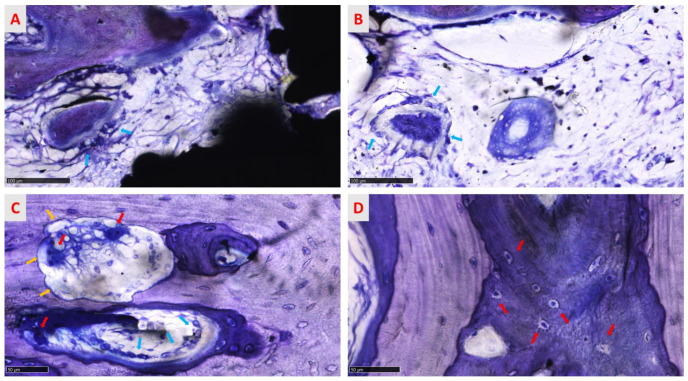
The panel highlights cell activity during the modeling and remodeling process. In (**A**) (chemically treated group) and (**B**) (untreated group), bony islands in the phase of formation and mineralization surrounded by osteoblast cells (light-blue arrows) are visible. In (**C**), a cutting cone is characterized by multinucleated cells (red arrows) housed in Howship lacunae (yellow arrows) and osteoblasts (light-blue arrows) that all together maintain the bone structure. (**D**) Osteocyte cells in different grades of maturation: the red arrow indicates less mature osteocytes housed in large lacunae, identified by the irregular shape, immersed in a violet matrix rich in collagen fibers. Close to the immature lacunae, mature tapered lacunae are visible, with osteocytes housed in calcified bone matrix (light brown). Toluidine blue/pyronin yellow, (**A**) total magnification 320×, (**B**) total magnification 320×, (**C**) total magnification 400×, and (**D**) total magnification 500×.

The osseointegration process is evidenced in Figure 9 and Figure 10, where the progressive growth of bone formation is visible, presenting different stages of calcification in all groups. No morphological differences were found between treated and native groups. In cortical bone, the process of osseointegration proceeded faster than in spongious bone but the defect was not entirely re-established (Figure 9A,B) in both treated and native groups 6 weeks after surgery. Different grades of mineralization are highlighted in shades of blue and violet, suggesting that the bone was not wholly mineralized and the calcification process proceeded from the circumferential to the inner implant portion (Figure 9A,B). Indeed, little areas inside the implant macropores were not completely regenerated, indicating that the process is actively ongoing. Many islands of new bone tissue sustained by a rich matrix of fibers are observable in the inner part of the site (Figure 12A).

Furthermore, in circumferential bone, some cutting cones were detected, indicating that surgery was involved in stimulating the modeling process (Figure 12 C). The spongious bone was characterized by new trabeculae deposed from the circumferential bone toward the inner portion of the randomized trabecular titanium structure. As found in cortical bone samples, some initial bony trabeculae resembling bony islands and surrounded by numerous osteoblasts were recognizable among new medullary spaces of regenerated area (Figure 12B). In spongious bone, morphological aspects revealed a physiological architecture characterized by a mesh of bone trabeculae enclosing wide medullary spaces rich in fibrovascular tissue and bone cells, suggesting that the bone is still evolving in an anabolic, depositional phase (Figure 11B). At all sites, numerous osteoblasts and osteocytes cells were visible. Osteoblasts presented a cuboid shape, indicating the active phase of collagen matrix deposition and matrix calcification. At the same time, osteocyte cells were observable and appear in Figure 12D in immature and mature phases, distinguishable by the shape of the lacunae.

#### 3.3.5. Semi-Quantitative Analysis of the Regenerated Area

The mean scores computed to describe the tissue composition in the regenerated area confirmed the qualitative morphological observation. More lamellar bone was present in the cortical bone than in the spongious bone. In the native group, both in cortical and spongious bone, the mineralization process led to a faster physiological decrease of woven bone and osteoid in favor of lamellar bone than in the treated group (Table 2).

## 4. Discussion

The long-term success of the titanium implants used to rehabilitate patients in orthopedics and dental-maxillofacial surgery depends on the interaction between the prosthesis and the bone. Both the shape and the surface of the titanium implants are considered decisive factors for achieving primary and secondary stability as they speed up osseointegration. [20,21,22,23,24]. AM technologies allow the realization of complex 3D structures with peculiar shapes, such as a trabecular structure, that improve osseointegration by mimicking the human bone architecture [20,25,26,27]. In brief, using the CAD/CAM procedure, starting from powder to produce multiform construction components through a stratum-layer strategy and a 3D CAD design [28,29], the “creation” of objects of complex architecture has been realized. Additional superficial modification of the trabecular implants could be effectuated using chemical agents or mechanical media that increase the micro-roughness to stimulate osteoconduction and accelerate prosthesis integration [20,23,24,30,31]. Tsukanaka and colleagues observed that compared to untreated surfaces, surface treatment (chemical and thermal etching) of trabecular titanium specimens by SLM technology significantly improves cell differentiation. In addition, the response of the native specimens was the same as that of the smooth surface titanium control specimens [15,16]. De Wild and colleagues demonstrated in vivo that SLM trabecular titanium specimens treated by sand-blasting and acidification exhibit greater osteoconductive properties than native specimens [17]. Xu et al. confirmed the results of the previous works, showing encouraging in vivo data in terms of osseointegration and bone regeneration for the trabecular titanium specimens obtained by SLM technology subjected to surface treatments by sand-blasting, followed by chemical treatment to remove the oxide layer, followed by acidification, when compared to native implants used as a control. However, the issue is still debated [18]. The current paper aimed to evaluate if the addition of a chemical treatment increasing the micro-roughness of the surface may lead to improved osseointegration of implants with a randomized trabecular titanium structure placed in an in vivo ovine animal model. Our results showed no differences among trabecular scaffolds with chemically treated and native surfaces. Irrespective of surfaces with micro-roughness, the trabecular structures studied here were designed to have the same features: a pore size distribution that promotes osteogenesis, capillary formation that provides a vascularized environment, and a porosity that mimics that of trabecular bone tissue. Moreover, in both groups, the tissue seemed well in contact with the implant surface inserted in the bone in press-fit. The tissue was at a high level of mineralization, and the osteoblasts cells were in the active phase of bone matrix deposition to surround and englobe the trabecular structure without gaps. The press-fit procedure seems to stimulate and improve osteoconduction of the cells, ensuring primary stability and favoring secondary stability because of a lack of micromovement that increases the timing of osteointegration [32]. The results of our study can be influenced by the press-fit achieved from the two types of implants, irrespective of chemical treatment. Effective bone ingrowth can be affected by the presence of fibrous tissue ingrowth. Fibrous tissue ingrowth was shown to be caused by micromotion of as little as 75 µm, so the press-fit of the implant is potentially critical to promote bone ingrowth [32]. The trabecular structures studied here have been designed to achieve an adequate press-fit regardless of surface treatment. Indeed, to obtain a strong press-fit, the randomized trabecular structures were supplied with randomized spiked trabeculae (diameter of 0.2 mm) outside the lateral surface of the cylinder (diameter 6 mm) that enveloped the implants. The design of fully trabecular cylindrical implants improved the press-fit and prevented micromotion and probably was the reason for the high level of osseointegration observed after 6 weeks irrespective of the chemical treatment of the surface. Using different porous structures (similar to the regular one) in a different animal model (rabbit model), other authors [17,18] have found an advantage in adding chemical surface treatment to the native surface of the porous structures obtained by AM. In reported studies, osteointegration was driven by a microscale surface roughness rather than by the design of the porous structure itself. In our study, the implants were optimized to achieve a strong press-fit and stability, confirming the appeal of new AM technologies that can be used to create new porous structure designs, such as a randomized trabecular structure, that can overcome the demand for additional chemical surface treatments. The results of our study confirm the role of achieving an adequate initial press-fit to obtain successful osseointegration of the implants. Furthermore, the chemical treatment requires further processing to manufacture the implants, which can compromise the mechanical properties of the structures themselves due to the removal of the titanium material. The biocompatibility of the titanium implants can also be affected by a standard and unsatisfactory cleaning process [33]. However, removal of the unmelting titanium powder particles is ensured by the sand-blasting process required to clean the implants manufactured by AM technologies. The current experimental study could open a new stage of clinical investigations. The evaluated devices, in fact, seem to be indicated in all clinical applications where having a smooth surface is an advantage. For example, a smooth 3D dental implant surface could aid in primary and secondary stability and, at the same time, reduce the risk of bacterial infiltration in the nano micropore of the surface, which can lead to the loss of the osseointegrated implant. In conclusion, the proposed structure seems efficient without superficial micro modification, positively impacting manufacturing costs and timing. The high impact of the time consumption of the industrial process can be reduced since the trabecular structure is osteoconductive, improving cell adhesion and osteointegration without surface modification.

## Figures and Tables

**Table 1:** Bone-to-implant contact (BIC ) and bone in-You growth (Bin) mean and standard deviation.

	6 Weeks			
Bone	BIC		BIn	
	Mean	SD	Mean	SD
Cortical Bone				
Treated	74.79%	16.92%	73.72%	11.47%
Native	77.10%	16.21%	81.58%	9.87%
Spongious Bone				
Treated	27.20%	3.84%	29.25%	3.88%
Native	30.43%	9.80%	33.53%	20.05%

**Table 2:** Score mean values for each tissue volume fraction for each experimental group.

	Lamellar Bone	Woven Bone	Osteoid	Soft Tissue
Cortical Bone				
Treated	3.63	1.5	1.13	1.38
Native	3.83	1.5	1,00	1.13
Spongious Bone				
Treated	1.13	1.25	2.38	3.00
Native	1.63	1.5	1.63	3.38

## Data Availability

Data are available upon request to the corresponding authors.
